# 96. *Candida albicans* bloodstream infections are comprised of diverse populations of strains, including antifungal tolerant strains that emerge during treatment failure

**DOI:** 10.1093/ofid/ofae631.033

**Published:** 2025-01-29

**Authors:** Cornelius J Clancy, Shaoji Cheng, Giuseppe Fleres, Minh-Hong Nguyen

**Affiliations:** University of Pittsburgh, Pittsburgh, Pennsylvania; University of Pittsburgh, Pittsburgh, Pennsylvania; University of Pittsburgh, Pittsburgh, Pennsylvania; University of Pittsburgh, Pittsburgh, Pennsylvania

## Abstract

**Background:**

The longstanding model is that most bloodstream infections (BSIs) are caused by a single strain that passes through a bottleneck. *C. albicans* exhibits genomic and phenotypic plasticity, but it is unknown whether within-patient (pt) strain diversity is common during BSIs.

**Figure 1.**
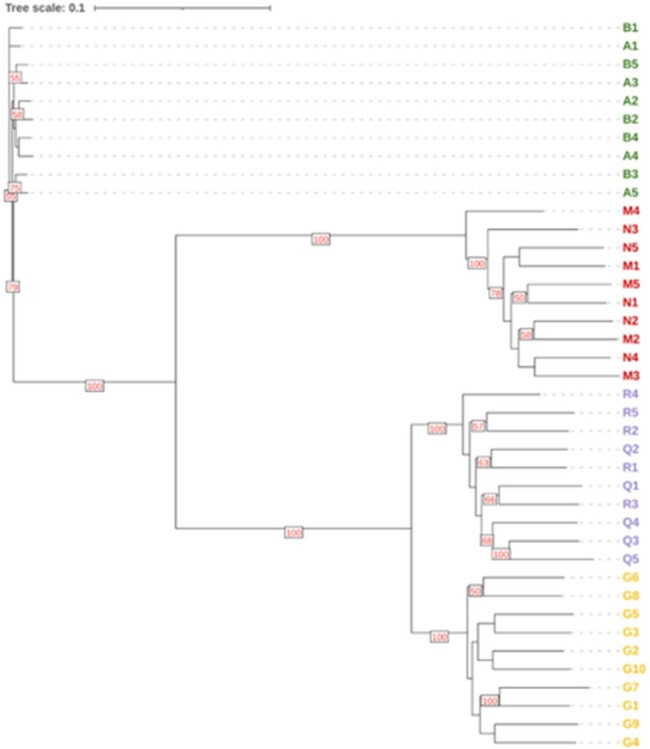
*C. albicans* SNP phylogeny Strains within each patient's blood cultures differed by SNPs and indels

**Methods:**

We determined short-read whole genome sequences (Illumina NextSeq) of 10 *C. albicans* strains from blood cultures (BCs) in each of 4 pts at baseline (*n*=4) and during persistent BSIs (3 of 4). In depth phenotypic assays were performed on strains from 1 pt.

**Figure 2.**
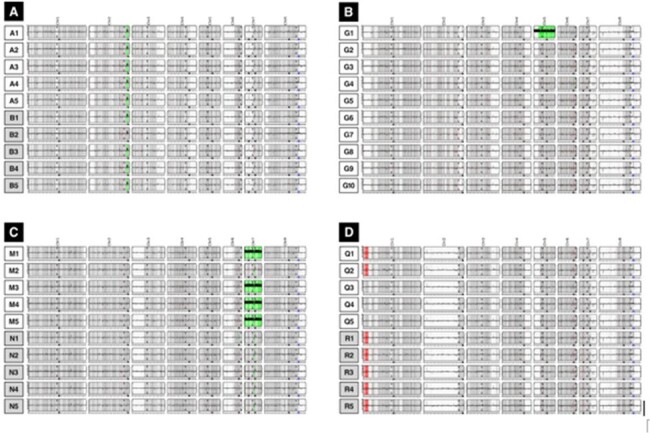
*C. albicans* large scale genomic variants Strains within 3/4 pts (MN, QR, G) were distinct by large scale variants, including aneuploidies, copy number variations and LOH.

**Results:**

Strains clustered by pt by single nucleotide polymorphism (SNP) phylogeny. BCs from each pt contained mixed populations of strains that differed by SNPs and indels (*n*=4 pts; 0-42 SNPs, 0-9 indels/pt), gene copy numbers (*n*=2), chromosome 5 or 7 aneuploidy (*n*=2), and loss of heterozygosity (*n*=1) [Fig 1, 2]. Chromosome 7 trisomy (Tri7) and euploid strains from 1 pt (MN) exhibited identical antifungal minimum inhibitory concentrations. However, euploid strains significantly outcompeted Tri7 strains in presence of micafungin *in vitro*, consistent with echinocandin tolerance [Fig 3]. In absence of micafungin, Tri7 strains significantly outcompeted euploid strains within human blood *ex vivo* and during mouse gastrointestinal colonization, colon invasion and BSIs, despite attenuated hyphal formation *in vitro* and *in vivo* [Fig 4]. Over-expression of chromosome 7 gene *NRG1* (transcriptional repressor) in euploid strains recapitulated Tri7 phenotypes, including repressed hyphal and biofilm formation. Tri7 strains represented 76% of the baseline BC population in pt MN. After 2 days of micafungin treatment, euploid strains were ≥ 95% of the population. In control experiments, changes in ploidy were not evident following incubation of index strain M1 in BC bottles *in vitro*.

**Figure 3.**
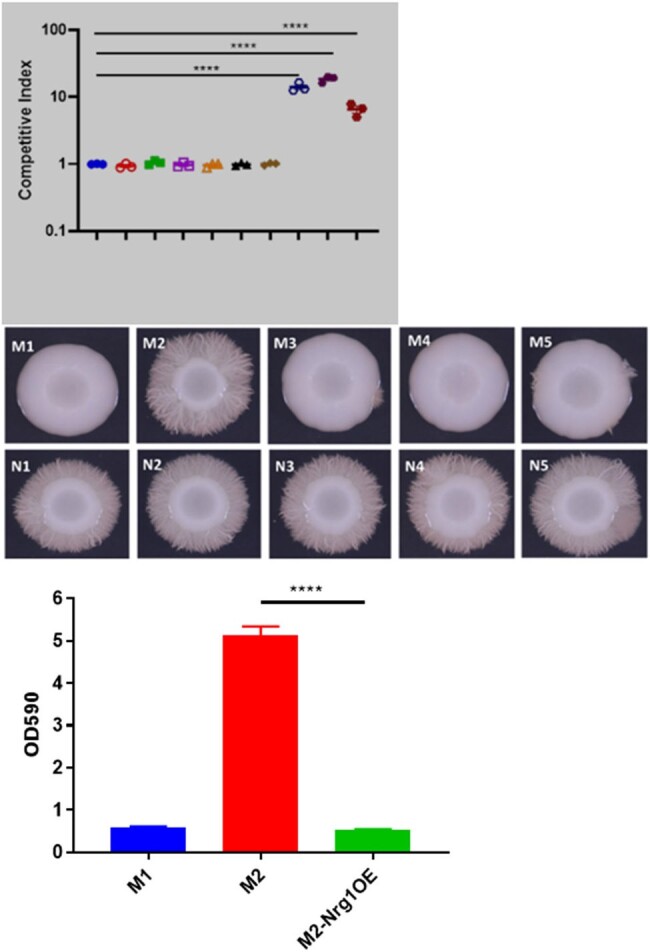
Phenotypic differences between Tri7 and euploid strains from pt MN A) M2 (euploid) outcompetes M1 (Tri7) in presence of micafungin in vitro in absence of differences in MIC. B) Tri7 strains are attenuated in hyphal formation. C) NRG1 overexpression in euploid M2 background represses biofilm and hyphal formation.

**Conclusion:**

Positive BCs are comprised of diverse *C. albicans* populations rather than single, genetically identical strains. Within-pt strains may demonstrate differences in responsiveness to antifungal agents and virulence that are not recognized by the clinical micro lab. In some cases, antifungal treatment failures are due to selection of pre-existing resistant or tolerant strains. Results challenge standard clinical and microbiology practices that focus on single *Candida* strains.

**Figure 4.**
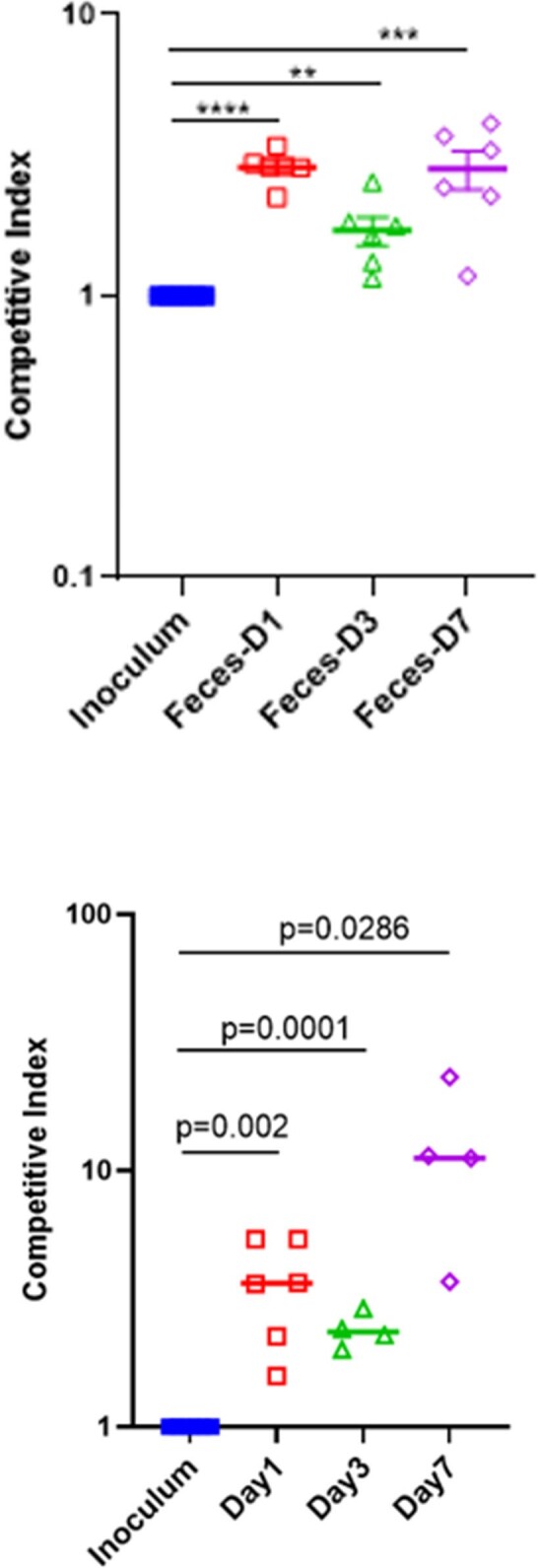
Tri7 strain M1 outcompetes euploid strain M2 during GI colonization and disseminated candidiasis. A) GI colonization mouse model. B) Competitive indices within kidneys during disseminated candidiasis

**Disclosures:**

**Cornelius J. Clancy, MD**, Cidara: Grant/Research Support|Gilead: Honoraria|Merck: Grant/Research Support|Scynexis: Advisor/Consultant|Shionogi: Advisor/Consultant|Venatorx: Advisor/Consultant

